# Attitudes of medical students towards incentives offered by pharmaceutical companies- perspective from a developing nation- a cross sectional study

**DOI:** 10.1186/1472-6939-15-36

**Published:** 2014-05-05

**Authors:** Usman Tariq Siddiqui, Amarah Shakoor, Sarah Kiani, Farwa Ali, Maryam Sharif, Arun Kumar, Qasim Raza, Naseer Khan, Sardar Mohammed Alamzaib, Syed Farid-ul-Husnain

**Affiliations:** 1 Medical student, Medical College, Aga Khan Medical University Hospital, P.O Box 3500, Stadium Road, Karachi, Pakistan; 2 Department of Community Health Sciences, Aga Khan Medical University Hospital, Karachi, Pakistan

**Keywords:** Pharmaceutical companies, Acceptability, Incentives, Attitudes, Medical students

## Abstract

**Background:**

A training physician has his first interaction with a pharmaceutical representative during medical school. Medical students are often provided with small gifts such as pens, calendars and books, as well as free lunches as part of drug promotion offers. Ethical impact of these transactions as perceived by young medical students has not been investigated in Pakistan before. This study aimed to assess the association of socio-demographic variables with the attitudes of medical students towards pharmaceutical companies and their incentives.

**Methods:**

As part of a cross-sectional survey, a validated questionnaire previously used for assessing attitude of medical students towards pharmaceutical industry, was modified, pre-tested and distributed among consenting clinical year students at DUHS and AKU. Questions included acceptability of pharmaceutically sponsored gifts, events and tuition fee, and their impact on future prescription. Responses were graded as agree, disagree or neutral which were then scored according to the AMSA guidelines of ethical conduct.

**Results:**

Out of a total of 353 targeted students 303 responded, corresponding to a response rate of 85.8%. Responses indicated that 42.7% students believed in no interaction with drug companies during medical school. However, 81% of students favored pharmaceutical sponsorship of student-body events/seminars at medical colleges. More than one-third of the students were comfortable receiving gifts from drug companies. Overall, the results of this study offer an interesting comparison between the students of a private medical school (AKU) and a public medical school (DUHS); AKU students exhibited a greater degree of mistrust towards drug information provided by pharmaceutical companies compared to DUHS students (p = 0.040). Furthermore, when asked if there was a need to incorporate guidelines in the undergraduate curriculum with regard to interaction with drug companies, 84.2% students at AKU agreed, compared to 54.9% at DUHS. Medical student Attitude Scores are more or less similar to each other independent of their various demographical differences.

**Conclusion:**

This study highlights that medical students in our population have a high level of acceptability towards incentives offered by pharmaceutical industry and that formal guidance regarding the subject should be incorporated into medical curriculum.

## Background

The importance of the relationship of a practicing physician with pharmaceutical industry and its representatives is irrefutable. According to previous estimates, 85-90% of medical doctors in US, Canada, Britain and New Zealand meet with pharmaceutical representatives (PR) [1]. PRs are drug marketing personals who approach physicians, present research and provide incentives, all of which is aimed towards convincing the physician to prescribe their brand. Incentives include drug promotional offers and also free samples, gifts and sponsorship of conferences among others. At this point, three key factors need to be considered: the appropriateness of accepting these incentives, favoring a particular company based on such incentives, and being able to objectively scrutinize the worth of the drug as well as that of the research being presented to support it.

The concern for drug company-physician relationship and its negative influence on prescription writing, has led numerous professional organizations, such as the American Medical Association (AMA) and the American Medical Student Association (AMSA), to develop guidelines and recommendations. AMSA recommendations discourage physicians and students from accepting gifts from drug companies, urge hospitals and residency programs to discontinue drug company-funded lectures and lunches, and oppose continuing medical education (CME) granted credit for drug company-sponsored events [2].

In this regard, AMSA initiated its PharmFree campaign in 2002 and released a policy statement regarding best practice aimed for the benefit of both practicing and training physicians. This policy aims to guide medical doctors and students towards ethically sound attitudes that should be fostered towards pharmaceutical industry sponsored gifts and meals, free pharmaceutical samples, pharmaceutical representatives, conferences and “indulgence” of pharma industry in medical student education and training. An excerpt from the current AMSA pharma-free policy is as below:

1. **Gifts and Meals:***Best Practice:* All gifts and on-site meals funded by industry are prohibited, regardless of nature or value.

2. **Pharmaceutical Samples:***Best Practice:* Industry samples are prohibited, except under certain narrow circumstances approved by the institution that protect the interests of indigent patients and prevent the use of samples as a marketing tool.

3. **Drug Representatives:***Best Practice:* Pharmaceutical and device representatives are not allowed to market their products anywhere inside the medical center and associated clinics and offices.

4. **On-site Educational Activities:***Best Practice:* Industry is not permitted to provide direct financial support for educational activities, including CME, directly or through a subsidiary agency.

5. **Compensation for Travel or Attendance at Off-site Lectures & Meetings:***Best Practice:* Personnel may not accept payment, gifts or financial support from industry to attend lectures and meetings.

6. **Industry Support for Scholarships & Funds for Trainees:***Best Practice:* The policy must either prevent industry from earmarking or awarding funds to support the training of particular individuals.

7. **Medical school curriculum (or other documentation of educational objectives/course content):***Best Practice:* Students are to be trained to understand institutional conflict-of-interest policies and recognize how industry promotion can influence clinical judgment.

One must realize that the very first interaction that a physician-in-training has with a pharmaceutical representative is during the early years of medical school [3-5]. In many instances, medical students are provided with small gifts as pens, calendars and books, as well as free lunches as part of drug promotion offers [6]. Studies at various US and Canadian medical schools have found that students receive many such gifts and their interaction with PRs is quite common; some studies have shown this interaction to be as frequent as ten times per month [3,7-11]. Additional studies have suggested that medical students tend to favor these interactions (3–7). Comparable results were demonstrated in a recent study from India [11]. This has led many critics to believe that these subliminal marketing strategies affect prescription-writing behavior when these students enter the professional world (8, 11). However, literature assessing medical students’ attitudes and exposures to the pharmaceutical industry and the marketing tactics deployed by them is quite sparse. Available studies report that most students believe that they are not much affected by the marketing schemes used by the pharmaceutical companies [12,13]. Many regard receiving trivial gifts from the pharmaceutical companies as ethically correct. For instance, one study found that although 85% of medical students believed it to be highly improper for a politician to receive a $50 gift, however receiving a similar gift from a pharmaceutical representative themselves was regarded as unsuitable by only 46% of students (10). Nevertheless, many have sensed the serious lack of training at undergraduate level pertaining to student interactions with PRs [10]. Substantial deficits have been found in students’ knowledge about pharmaceutical marketing expenditures, professional ethics and accuracy of drug information from pharmaceutical representatives [5,10].

Strong evidence suggests that medical students are at-risk of being influenced by pharmaceutical company marketing strategies in the form of free gifts [5,14,15]. Students who receive gifts may believe that they are receiving something for nothing, contributing to a sense of entitlement which is not in the best interests of their moral development as future doctors. Alternatively, students may be subject to recognized or unrecognized reciprocal obligations that potentially influence their decision making [16]. A randomized controlled experiment by Grand D et al. validated that subtle exposure to even small pharmaceutical promotional items influences implicit attitudes towards marketed products among medical students [10].

According to the Pakistan Medical and Dental Council (PMDC), the number of medical schools in Pakistan has considerably increased in the past 15 years from a mere 30 in the year 2000 to almost 90 in 2013. Out of these, 38 are public and 52 are private institutions. The structure of medical education in Pakistan varies considerably in terms of teaching methods. However, all programs consist of a 5-year rigorous training curriculum during medical school. This is usually followed by relevant specialty training/residency. Medical college falls under a specific university in its undergraduate medical education (UGME) program. Each university also usually has its own residency programs for multiple specialties under its postgraduate medical education (PGME) program.

A literature search was conducted on Pubmed/Medline using the keywords “attitudes”, “medical students”, “pharmaceutical industry”, “Education, Medical, Undergraduate” and “Pakistan” in different combinations to identify studies from Pakistan. To the best of our knowledge, we found no similar studies on the presented topic. This in itself enhances the need for conducting such a study in Pakistan. We, as medical students, have observed that PRs approach students in pharmaceutical exhibitions held within college premises, at medical conferences and also during outpatient clinics. Often at times, there is an exchange of small gift items and free meals. Ethical impact of these transactions as perceived by young medical students has not been investigated in Pakistan before.

Through this study, we aim to assess the attitude of medical students towards: a) gifts and sponsorship offered by pharmaceutical companies, b) effects of pharmaceutical marketing on prescribing behavior of physicians, c) interaction between pharmaceutical companies and healthcare professionals. We also aim to investigate the differences of opinions between medical students from public-sectored and private institutes. Finally, we aim to assess the association between socio-demographic variables and the subsequent attitude of medical students towards pharmaceutical companies and their incentives. To the best of our knowledge, this study is the first attempt by any authority to gauge not only the attitudes and perceptions of medical students regarding the pharmaceutical industry, but also to identify the socio-demographic patterns related to such thinking.

## Methods

This is a cross-sectional study carried out at two medical colleges in Karachi, Pakistan. Aga Khan University (AKU) Medical College was chosen to represent the largest private medical set-up in the country with an average batch of 100 students and Dow University of Health Sciences (DUHS) representing a leading public sector medical institution with an average class size of 200 students. Karachi is the largest cosmopolitan city of Pakistan. This was a favorable factor given the city’s diverse population belonging to different social strata. Convenience sampling was employed. Sample size was calculated assuming the proportion of favorable attitudes towards pharmaceuticals to be 50%. A confidence interval of 95% and bound on error of 5% were utilized. Open Epi was used to calculate the sample size which was 282. Assuming a predicted response rate of 80%, the adjusted sample size came out to be 353 individuals.

All male and female medical students, currently in their clinical years (which correspond to the third, fourth and fifth year of the Bachelor of Medicine & Bachelor of Surgery [MBBS] program), formally enrolled at AKU or DUHS medical college were included. All students involved in the study design or conduction in any capacity, and those students on whom the questionnaire was pre-tested were excluded from the study.

The definition of attitude was taken as suggested in the oxford dictionary; ‘a settled way of thinking or feeling reflected in a person’s behavior’. In sociology attitude is taken to be an orientation (towards a person, situation, institution, or social process) which is indicative of an underlying value or belief.

In order to assess the ‘attitude’ of medical students towards the pharmaceutical industry in our setting, a validated questionnaire suited for medical students was adapted from a similar study by Joseph Barfett et al. [17] (Attached as Additional file 1). With the help of this questionnaire, we evaluated the willingness of medical students to have any form of interaction with pharmaceutical representatives and their perceived appropriateness of accepting various ‘gifts and favors’ from pharmaceutical industry.

Modifications in the questionnaire made for this study included the conversion of Dollars ($) to Pakistani Rupee (Rs.) in the question regarding parental income. Two supplementary open-ended questions were included which enquired about the form of individual interaction with pharmaceutical industry and its influence on the students. These additions were deemed necessary because the issue at hand is relatively under-investigated in our setting and the exact manner of student interaction with pharmaceutical representatives and its impact has not been well-characterized. The modified version of this questionnaire was pre-tested before being administered to actual participants. This was done by directing the questionnaire to a select group comprising of fifty medical students from Aga Khan Medical College, who were not involved in the actual study later. During pre-testing, difficulty with questions, instructions, order of questions or responses was considered, and the time taken to fully answer the questionnaire was duly recorded. Questions were assessed to be unambiguous and socially apt; no problems were identified and hence, no further modifications were required.

The questionnaire comprised of nineteen questions and was divided into two parts. In order to maintain participant confidentiality, no personal information was sought. The first part dealt with five questions pertaining to the study populations’ demographic and socioeconomic details. Students were asked to give information regarding their sex, year of study, average monthly parental income, and parent’s profession as a physician or belonged to the pharmaceutical industry. The second part of the questionnaire consisted of fourteen questions evaluating the medical students’ views towards interaction with pharmaceutical representatives and the incentives they offered. This was done with the help of hypothetical scenarios and relevant inquiry about their potential attitudes in such situations. These questions were grouped into three categories for analysis of responses; attitude regarding receiving gifts from pharmaceutical representatives, event and tuition fee sponsorship by pharmaceutical industry and the subsequent impact of these incentives on prescription preferences later during practice. Nine of the fourteen questions consisted of pre-defined responses of ‘Agree’, ‘Neutral’, and ‘Disagree’. Multiple choice answers were offered to three questions. The final part of the questionnaire consisted of two open ended questions where students were asked about their views relating to student-industry interactions and to specify if any particular experience relating to such interactions had influenced their responses to the entire questionnaire. As no legal rules and regulations exist in our medical setting regarding interactions between the pharmaceutical industry and medical students, AMSA guidelines were followed in order to define inappropriate and appropriate attitude.

A cumulative score was calculated as an overall portrayal of medical student’s attitudes; this new attitude scoring (ATT Score) system was developed depending on the respondents’ answers to the individual questions in the questionnaire and has not been used before. Students demonstrating a compliant attitude towards pharmaceutical companies were given a higher score and those displaying reservations towards the same were given lower scores. Answers revealing favorable attitudes towards medical student-PR interactions were given a score of 3, all neutral responses were given a score of 2 and student responses showing strict attitudes towards drug companies were given a score of 1. Cumulative scores for each respondent were then calculated (ATT Score)

The study protocol was approved by the Ethics Review Committee of the Aga Khan University Hospital. The consent form was approved in both English and Urdu languages. In order to ensure preservation of meaning, Urdu consent form was translated back into English. Individual permission to conduct survey was sought from the respective deans of AKU and DUHS medical colleges. Both male and female students belonging to clinical years were approached in the medical college courtyard at both institutions, during break hours. Background, purpose and significance of the study were explained to the participants, and consent was sought before enrollment. All consenting participants were requested to fill out the questionnaires. They were given ample time to respond in privacy to ensure confidentiality and freedom of response without fear of judgment. No attempt was made by the persons administering the questionnaires to interpret the questions, or influence the participant’s ideas and responses. After completion, filled forms were collected and kept under safe custody for entry and analysis. To minimize contamination, data collection from DUHS medical college was completed in 2 days and from AKU medical college over the subsequent two days.

### Statistical analysis

Data was entered using Epidata software version 3.1 and was analyzed using SPSS version 17.0. Simple frequencies were run for individual questions. An independent t-test was used to compare scores in between groups of students belonging to different demographic categories i.e. gender, medical school, year of study, monthly parental income or parents belonging to medical or pharmaceutical professions.

## Results

### Demographics

Out of a total of 353 potential medical students, 303 responded, leading to an overall response rate of 85.8%. Among these, 102 were males and 201 were females. It must also be noted that response rate of each question varied. Representation from the two medical schools was not uniform; 38.3% (114) of the respondents were from AKU and the remaining 62.7% (184) belonged to DUHS Medical College. With reference to the participants’ parents, only 3% had either or both parents working for pharmaceutical companies, while 27.1% had either or both parents working as doctors. Tables 1 and 2 depict the demographics for the respondents in the study.

**Table 1:** Demographic distribution of the study respondents

**Demographic feature**	**Quantity (%)**
Sex	
Male	102 (33.7)
Female	201 (66.3)
Medical college	
AKU	114 (38.3)
DUHS	184 (61.7)
Clinical year	
3^rd^	93 (31.2)
4^th^	132 (44.3)
5^th^	73 (24.5)

**Table 2:** Difference in the net monthly parental income of the two study group respondents

**Parents’ income**	**AKUH**	**DUHS**
< 50,000 PKR	4 (3.7%)	38 (22.2%)
50,000 - 100,000 PKR	14 (13.0%)	64(37.4%)
100,000 - 150,000 PKR	13 (12.0%)	28 (16.4%)
150,000 - 200,000 PKR	18 (16.7%)	15 (8.8%)
> 200,000 PKR	59 (54.6%)	26 (15.2%)

### Level of acceptability towards interacting and receiving gifts from pharmaceutical companies

**Figure 1:**
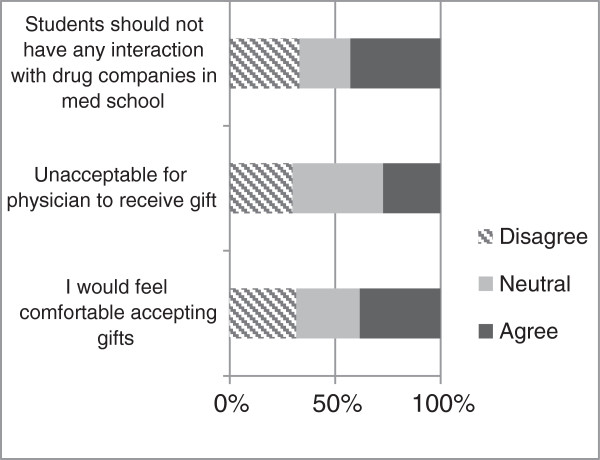
Attitudes of students on the acceptability of gifts from drug companies.

**Figure 2:**
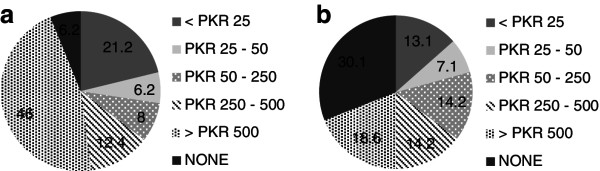
**Acceptability of gifts with monetary value. a.** AKU students. **b.** DUHS students.

Figure 1 showcases the results with regard to whether or not students should interact with, and in turn, receive gifts from pharmaceutical companies. These responses indicate that 42.7% students believe that there should be no interaction with drug companies during medical school. When asked if it was unacceptable for a physician to receive any gift from a drug company, similar numbers of students were neutral towards the notion. Furthermore, when asked whether they themselves would accept gifts, more than one-third of the students were comfortable with receiving certain named gifts. Only 30% believed that they would not accept gifts worth any monetary value (Figure 2). Figures 2a and 2b provide a contrast between AKU and DUHS students with reference to the inclination of students from both universities towards such gifts. It is important to note however that while results show a generally high level of acceptance of gifts overall, only 19-24% of respondents thought they would preferentially prescribe a drug that was marketed to them (Table 3).

**Table 3:** Comparison between the responses of students from AKUH and DUHS

**Question**	**Aga Khan university**			**DUHS Medical college**			
	**Disagree (%)**	**Neutral (%)**	**Agree (%)**	**Disagree (%)**	**Neutral (%)**	**Agree (%)**	**P-value**^**1**^
It is unacceptable for a physician to receive a gift from a drug company in any form	34.2	40.4	25.4	26.9	44.0	29.1	0.443
I would feel comfortable receiving the following gifts from a pharmaceutical company: lunch, palm pilot, penlight, stethoscope, textbook, watch/jewellery	30.7	28.9	40.4	31.9	31.9	36.3	0.834
I would preferentially prescribe a drug from one of the companies that provided me with gifts or incentives	56.1	19.3	24.6	56.5	24.5	19.0	0.429
Students should not have any interaction with drug companies in medical school	33.6	20.4	46.0	32.1	26.6	41.3	0.457
The information provided about drug effectiveness from pharmaceutical companies is untrustworthy	15.8	43.9	40.4	41.8	44.0	14.1	<0.05*
It is acceptable for physicians to be compensated PKR 100 by the drug company each time their drug is prescribed	78.9	5.3	15.8	60.3	30.4	9.2	<0.001*
It is acceptable for drug companies to sponsor events/educational seminars during medical school	11.4	8.8	79.8	7.6	9.2	83.2	0.597
If a drug company agreed to pay for the printing cost of all my class notes in undergraduate medical school, I would not mind the logo of that company appearing in the bottom corner of the first slide of my lectures	34.2	16.7	49.1	44.0	16.8	39.1	0.175
Do you feel that there is a need for incorporating guidance regarding relationship between the pharmaceutical industry and the medical professionals in the undergraduate curriculum	4.4	11.4	84.2	14.1	31.0	54.9	<0.001*

^1^p-values calculated are for inter-medical college difference *statistically significant.

One of the most contrasting finding between AKU and DUHS students was between the level of trust regarding information provided by pharmaceutical companies about the drugs. Almost 41% of AKU students considered information provided by pharmaceutical companies to be untrustworthy compared to a mere 14% by DUHS students (p-value <0.05) (Table 3).

### Prescription of drugs in return for compensation

**Figure 3:**
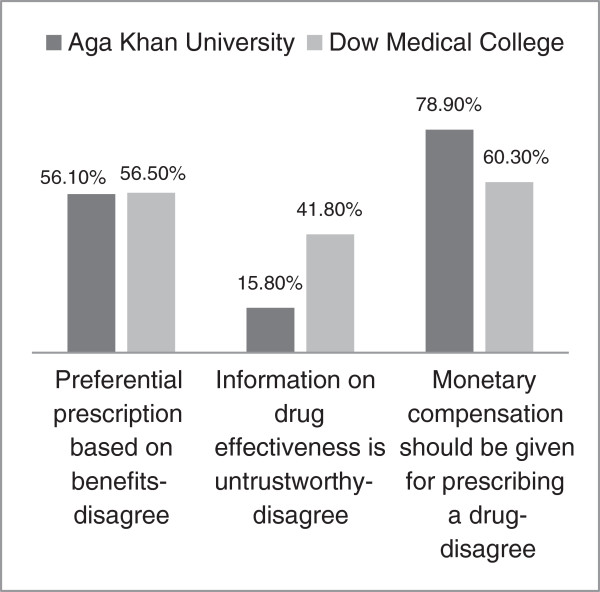
Comparison of AKU and DUHS attitude on preferential prescription.

Figure 3 shows data on some questions comparing the two universities sampled. When asked if they would preferentially prescribe drugs based on benefits they received from companies, more than 50% students disagreed, from both institutes. However, AKU students showed a greater degree of mistrust towards drug information provided by pharmaceutical companies compared to DUHS students (p-value <0.05). Furthermore, 78.3% of AKU students felt that physicians should not be compensated financially each time they prescribe a particular company’s product, compared to 60.3% students at DUHS who felt the same way (p-value <0.001).

### Pharmaceutical companies and their possible role in student life

A number of questions addressed the possible role that drug companies could directly play in student-life; for example, students were asked if it was acceptable for drug companies to sponsor events/seminars at medical colleges. All 9 students whose parent(s) worked for pharmaceutical companies agreed to accept such sponsorships, compared to 81% in the rest of the sample population. Furthermore, 45.1% students whose parent(s) were doctors were willing to accept the logo of a drug company in their lectures, if the company paid for the cost of lecture notes, compared to 42.1% in the rest of the sample though the results were not statistically significant. Additionally, majority of the students were prepared to accept tuition fee coverage from drug companies in return for attending their seminar. Notably, around 36% of the respondents from AKU and 26% from DUHS were comfortable receiving more than 30% tuition costs from pharmaceutical companies.

### Guidelines on interaction with pharmaceutical companies

When enquired about their preformed notions about pharmaceutical companies, more than 60% students agreed with the statement that these companies are primarily interested in profit; however they still try to work in the best interest of doctors and patients. For both AKU and DUHS, final year medical students picked this statement more frequently as compared to 3^rd^ or 4^th^ year students.

Additionally, when asked if there was a need to incorporate guidelines in the undergraduate curriculum with regard to interaction with drug companies, 84.2% students at AKU agreed to it, compared to 54.9% at DUHS (p-value <0.001). Table 3 showcases these results broadly.

### Attitude score

The attitude score was analyzed by making various subgroups of the respondents, though this was not planned initially. Scores from none of these subgroups appeared to be significantly different from one another as revealed (Table 4). One can thus gather from the above findings that medical student attitudes are more or less similar to each other regardless of their socio-demographical differences ***.*** Interestingly, the Attitude scores of AKUH and DUHS students were also similar.

**Table 4:** Attitude score for demographic variables

**Demographic variables**	**Option (n)**	**ATT Score (Mean** + **Std dev)**	**P-value**
Medical college	AKUH (110) DUHS (183)	24.3 + 5.9 24.1 + 4.7	0.879
Gender	Male (97) Female (196)	24.9 + 5.2 23.8 + 5.1	0.073
Year of study	3^rd^ year (91) 4^th^ year (128) 5^th^ year (69)	23.7 + 5.3 24.8 + 4.8 23.5 + 5.6	0.128
Parent medical doctor	Yes (79) No (214)	24.4 + 4.8 24.1 + 5.3	0.731
Parent in the pharmaceutical industry	Yes (9) No (284)	25.8 + 2.8 24.1 + 5.3	0.355

## Discussion

Literature suggests that a physician’s interactions with pharmaceutical representatives generally begin during medical school [18]. A vast majority of students are exposed to drug-marketing and may become more vulnerable to it. Medical students are targeted deliberately by pharmaceutical companies [4]. Most of these studies have been conducted in developed countries, whereas literature from the developing nations remains deficient (3–5, 20). Student attitudes toward interactions with pharmaceutical companies reveal the need for further education and guidance [19]. The results of our study displayed that students’ understanding about the system was unclear, at best. A large number of students were unsure about the appropriateness for doctors and medical students to receive gifts from pharmaceutical companies.

Evidence regarding the effect of the length of clinical training on students’ opinions regarding pharmaceutical incentives is contradictory. Most studies have concluded that the level of exposure to pharmaceutical representatives and their incentives differs between clinical and pre-clinical years, as does the acceptability of these gifts and offers [5,13,20]. To go a step further, we investigated the differences in responses among clinical students enrolled in three different years of training. Furthermore, we studied this effect separately for the DUHS Medical College and AKU in order to get a representation of the differences in opinions that exist between students enrolled in public and private sector institutes, respectively.

Our study validates the observation that the year of clinical clerkship indeed has an effect on the student’s approach towards the pharmaceutical incentives. This difference within the clinical years was more pronounced for the Aga Khan University: senior medical students from Year 5 of medical training were more acceptable of such gifts than students enrolled in clinical Years 3 and 4. Comparing students from AKUH and DUHS, we found that final year students from AKU were more prejudiced in their responses than the final year students of DUHS. The difference in the private and public sector was highlighted even further when it was found that AKU students were more comfortable with accepting expensive gifts than were DUHS colleagues. This may be due to a difference in the level of exposure of the two groups to pharmaceutical marketing [5,13]. Secondly, the net monthly parental income differed significantly between the two groups, which may directly account for the students’ enrollment in the respective medical colleges (Table 2); it maybe likely that difference in the socio-economic back ground of the students may account for this finding considering that most students at private university were relatively from a higher-income family background.

In Pakistan, there is no mechanism to monitor the drug promotional campaign by pharmaceutical industry: the extent of physician-industry interactions appears to affect prescribing and professional behavior of physicians worldwide as evidenced by most studies conducted in the developed world [18]. Approximately 90% of the General Practitioners declare that drug promotion has definitely had an influence on their prescribing pattern [21]. In our study, students from private institution, despite being more receptive of expensive gifts offered by the drug companies, were more skeptical about the information presented to them by drug companies with almost 41% considering the information provided about drug effectiveness from pharmaceutical companies as untrustworthy; were less likely to be influenced by these marketing practices in terms of prescription practices as noted with low percentage of acceptance of monetary compensation (21% of AKU students) than their public medical college counterparts (39%), and therefore might be more aware of the ethical boundaries involving drug prescription though this needs to be investigated formally. The better understanding of AKU students might be attributed to the university hospital policies on doctor-drug company relationship that exist within AKU. Looking at the differences pointed out between the two medical colleges it can thus be suggested that the students’ increased acceptability of gifts can possibly stem from a lack of knowledge of appropriate Medical Student-Drug Company interaction. Incorporating guidance about this relationship into their undergraduate curriculum can be monumental in helping them establish ground rules and limitations to the acceptable degree of their interaction with the drug companies [18,22,23]. Also, limiting the contact with pharmaceutical representatives might improve future attitudes and behaviors of these budding physicians [24].

It is known that attending drug company-sponsored CME events and accepting funding for travel or lodging for educational symposia are associated with increased prescription rates of the sponsor's medication [18]. The role of pharmaceutical sponsorship in student life is in fact more controversial. Our study displayed that an overwhelming majority of students were in favor of pharmaceutical company sponsored events and educational seminars. Medical students are in a particularly interesting position with respect to interactions with pharmaceutical companies: technically, they are part of the general population who are free from direct marketing of pharmaceutical products. However, they are often targeted by marketing from pharmaceutical companies through sponsorship of medical student societies. Guidelines need to be established dictating the limitations and drawing out boundaries of the medical student-pharmaceutical company interaction so as to facilitate an interaction that suitably benefits both parties while staying within the ethical domain as described by various institutions like AMSA [2].

Interestingly, more AKU students (84%) felt it necessary to incorporate guidance regarding doctor- pharmaceutical company interaction in the undergraduate compared to DUHS Students (54%). These responses further solidify our claims that AKUH students appear to have a more definitive understanding and were more critical in their overall approach towards the issue. The fact that only half of the students enrolled in the public sector understand the significance of incorporating guidance for pharmaceutical company interactions with healthcare professionals, reiterates the need to include such guidance within the medical curriculum.

To comprehensively assess the relation of various demographic characteristics of medical student population and their responses, the Attitude Scoring System was devised. This is a comprehensive tool which helped us in assessing the attitude of students towards pharmaceutical companies and their incentives. The mean Attitude score of the medical students wandered between 23 and 24 (the ideal-based on the AMSA guidelines-being a score of 11). Our study showed no relation of the gender, socio economic background, parents’ profession as doctors or parents’ employment in a drug company to their attitude towards pharmaceutical company marketing and incentives. Interestingly, the Attitude scores of AKU and DUHS students were also similar, despite the individual differences noted in their pattern of responses. The reason for this was that AKU students demonstrated a greater acceptability towards gifts and favors as compared to DUHS students. However, as shown by our results, DUHS students had a less critical opinion towards the reliability of pharmaceutical information and were more willing to prescribe drugs based on the benefits offered to them during clinical practice. This balanced out their mean Attitude score. This displays a possible limitation of the Attitude scoring system: scoring alone is not sufficient in assessing the trends in thinking—it is simply a tool to assess the overall inclination of subjects towards pharmaceutical company incentives, though it may differ if this study can be expanded to more than a few medical colleges and is therefore may be a question for further study in different demographical settings.

### Limitations

Most of the associations shown by our study have insignificant p-values which may be accounted for by the small sample size. This resulted because of the overall small size of the class in the medical college. As this was the first study in Pakistan addressing this idea to the best of our knowledge, it provides a good foundation-stone by showing the high overall inclination of medical students towards pharmaceutical incentives and the corresponding need for guidance on the topic since the actual prevalence of these interactions in general among Pakistani medical students is unknown. Future studies with larger samples and participation of multiple medical schools are needed in order to clearly verify this phenomenon and elucidate factors predicting attitudes and future behaviors of medical students. There was also a lack of formal assessment of the level of awareness of institutional policies regarding the limitations of pharmaceutical interactions with physicians, a factor that might have offered answers to a few of the critical findings. Since this is a cross-sectional study, we cannot confidently comment on whether certain factors were temporally related to the development of particular attitudes in medical students, however an idea about its prevalence can be made. Prospective or intervention studies are required to clarify such concerns. Other factors like awareness of company logic/business world were also not investigated here.

Generalizability of results is another potential limitation that the investigators identified as the study was carried out at only two centers in one city of Pakistan and responses may differ across various cities. Large multi-center surveys would be help-full to build a complete picture of the attitudes of medical students across the country from a wide variety of different backgrounds. Contamination bias was another potential issue as the questionnaire was pretested in the same location as our study sample. In order to minimize contamination we attempted to complete the data collection as soon as possible taking a total of 2 days to complete the AKU sample where the questionnaire was also pretested.

## Conclusion and recommendations

The first interaction between the pharmaceutical industry and future health care professionals occurs during years of medical education. Literature has repeatedly shown the widespread interaction between medical students and pharmaceutical representatives, and a high level of acceptability towards their gifts and incentives, giving rise to a concern that whether such an early exposure will influence prescribing practices of future physicians and how this issue might be addressed. With a rapidly evolving pharmaceutical industry in the developing countries and young doctors joining the field of medicine there are increased chances of interaction in the absence of any medical ethics education on the subject of doctor-pharmaceutical interaction. Our study findings mirror those from other parts of the world. Students approached in our study belonging to both public and private setup had pre-existing opinions regarding appropriate doctor-pharmaceutical interaction and demonstrated a high level of acceptability towards incentives and gifts offered by them. This acceptability was not significantly correlated with socio-demographic variables which we investigated, but a significant difference existed between the public and private set-up possibly pointing to the fact that there might be some other factors in which medical students are trained which have a substantial impact on their professional ideologies and ethical standards. Based on some of these finding we can conclude that it is imperative that every medical college ideally incorporate guidance regarding doctor pharmaceutical interactions, practice clear ethical policies which medical students should be aware of, and help foster a supportive environment regarding such social issues so that our future physicians are more apt to handle them. Further studies need to be undertaken with a more diverse student sample and a larger sample size firstly to identify the prevelance rate of these interactions and secondly to clearly elucidate reasons that might predict factors affecting the opinions that students have towards the pharmaceutical industry and help institutions frame their mode of guidance regarding such issues in a more feasible way.

## Abbreviations

DUHS: Dow University of Health Sciences; AKUH: Aga Khan University Hospital.

## Competing interest

The authors have no financial disclosure. The authors have no conflict of interest.

## Authors’ contribution

US: Came up with the concept and participated in designing the proposal, performed the statistical analysis and participated in drafting the manuscript and revision, AS: Participated in designing the proposal, acquisition of data and drafting the manuscript, SK: Participated in acquiring data and drafting the manuscript, FA and MS: Participated in drafting the manuscript, AK, QR, NK and MA: Participated in acquiring data, FH: Supervised the project and provided critical analysis of the manuscript. All authors read and approved the final manuscript.

## Pre-publication history

The pre-publication history for this paper can be accessed here:

http://www.biomedcentral.com/1472-6939/15/36/prepub

## Supplementary Material

Additional file 1 Questionnaire. Click here for file
