# Supplementary material for: Attitudes of medical students towards incentives offered by pharmaceutical companies- perspective from a developing nation- a cross sectional study
Source: BMC Med Ethics. 2014 May 5;15:36. doi: 10.1186/1472-6939-15-36 (PMC4101871; doi:10.1186/1472-6939-15-36)
Supplement: Additional file 1 — Questionnaire. [file 1472-6939-15-36-S1.docx]

**Appendix 1**

**Serial ID____________**

Name (Optional)_________________________

**Demographics**

1. Sex
2. Male
3. Female
4. Educational Background
5. Arts
6. Commerce
7. Science
8. Year of study in the medical School
9. Year 3
10. Year 4
11. Year 5
12. Approximate parental income (monthly)
13. < PKR 50, 0000
14. PKR 50,000 – PKR 100000
15. PKR 100000 – PKR 150000
16. PKR 150000 – PKR 200000
17. > PKR 200000
18. Do you have any parent(s) who is a medical doctor?
19. Yes
20. No
21. Do you have at least one parent working for the pharmaceutical industry?
22. Yes
23. No

____________________________________________________________________________________

1. It is unacceptable for a physician to receive a gift from a drug company in any form.
2. Disagree
3. Neutral
4. Agree
5. I would feel comfortable receiving the following gifts from a pharmaceutical company: golf clubs, lunch, palm pilot, penlight, stethoscope, textbook, watch/jewellery.
6. Disagree
7. Neutral
8. Agree
9. I would feel comfortable accepting gifts from a pharmaceutical company worth
10. < PKR 25
11. PKR 25 - 50
12. PKR 50 - 250
13. PKR 250 - 500
14. > PKR 500
15. Five drugs from five different companies are identical in terms of price, efficacy and effectiveness. I would preferentially prescribe a drug from one of the companies that provided me with such gifts or incentives mentioned in question #2 and #3 over those from companies that did not.
16. Disagree
17. Neutral
18. Agree
19. Students should not have any interaction with drug companies in medical school.
20. Disagree
21. Neutral
22. Agree
23. The information provided about drug effectiveness from pharmaceutical companies is untrustworthy.
24. Disagree
25. Neutral
26. Agree
27. As long as their medications are accepted to be part of the standard care it is acceptable for physicians to be compensated PKR 100 by the drug company each time their drug is prescribed.
28. Disagree
29. Neutral
30. Agree
31. It is acceptable for drug companies to sponsor events/educational seminars during medical school.
32. Disagree
33. Neutral
34. Agree
35. If a drug company agreed to pay for the printing cost of all my class notes in undergraduate medical school, I would not mind the logo of that company appearing in the bottom corner of the first slide of the lecture.
36. Disagree
37. Neutral
38. Agree
39. A drug company wants to increase its visibility to the medical profession and has recently approached the medical school. They would like to provide a one-day seminar regarding their product at the end of the second year of medical school. In return, they are willing to pay for a fraction of the second year tuition for each student who attends their seminar. As a medical student faced with increasing tuition costs, I think that it would be fair if the pharmaceutical company pays this percentage of my second-year medical school tuition:
40. 1 – 10% b. 10 – 20% c. 20 - 30% d. more than 30%
41. Which of the following statements do you think is most true of drug companies:
42. They are fundamentally on the same side as doctors and patients and should be regarded as an important part of the health care system.
43. They are fundamentally interested in profit and never on the side of wither doctors or patients.
44. They are primarily interested in profit: however, they still try to work in the best interest of doctors and patients.
45. Do you feel that there is a need for incorporating guidance regarding relationship between the pharmaceutical industry and the medical professionals in the undergraduate curriculum?
46. Agree
47. Neutral
48. Disagree
49. Briefly, what are your views about interactions between medical students and the pharmaceutical company?

_____________________________________________________________________________________

_____________________________________________________________________________________

1. Have you had any interactions with the pharmaceutical industry in the past that might have influenced your responses to the above questions? If yes please specify :

_____________________________________________________________________________________

_____________________________________________________________________________________
